# CRISPR/Cas9 as an antiviral against Orthopoxviruses using an AAV vector

**DOI:** 10.1038/s41598-020-76449-9

**Published:** 2020-11-09

**Authors:** Cathryn M. Siegrist, Sean M. Kinahan, Taylor Settecerri, Adrienne C. Greene, Joshua L. Santarpia

**Affiliations:** 1 WMD Threats and Aerosol Science, Sandia National Laboratories, Albuquerque, NM USA; 2 University of Nebraska Medical Center, Omaha, NE USA; 3 CWMD Research, National Strategic Research Institute, Albuquerque, NM USA; 4 TCR Discovery, Gritstone Oncology, Inc., Cambridge, MA USA

**Keywords:** Pox virus, Antivirals

## Abstract

A vaccine for smallpox is no longer administered to the general public, and there is no proven, safe treatment specific to poxvirus infections, leaving people susceptible to infections by smallpox and other zoonotic Orthopoxviruses such as monkeypox. Using vaccinia virus (VACV) as a model organism for other Orthopoxviruses, CRISPR–Cas9 technology was used to target three essential genes that are conserved across the genus, including A17L, E3L, and I2L. Three individual single guide RNAs (sgRNAs) were designed per gene to facilitate redundancy in rendering the genes inactive, thereby reducing the reproduction of the virus. The efficacy of the CRISPR targets was tested by transfecting human embryonic kidney (HEK293) cells with plasmids encoding both SaCas9 and an individual sgRNA. This resulted in a reduction of VACV titer by up to 93.19% per target. Following the verification of CRISPR targets, safe and targeted delivery of the VACV CRISPR antivirals was tested using adeno-associated virus (AAV) as a packaging vector for both SaCas9 and sgRNA. Similarly, AAV delivery of the CRISPR antivirals resulted in a reduction of viral titer by up to 92.97% for an individual target. Overall, we have identified highly specific CRISPR targets that significantly reduce VACV titer as well as an appropriate vector for delivering these CRISPR antiviral components to host cells in vitro.

## Introduction

Following the eradication of smallpox in 1977, the administration of vaccines for poxviruses has ceased, making humans susceptible to infections by these pathogens^1^. While other human poxvirus infections, such as those from vaccinia and cowpox, are often relatively benign in most immunocompetent individuals, monkeypox infections cause higher levels of mortality and morbidity^2^. This virus is primarily indigenous to Africa; however, studies suggest an increasing host range, virulence, and number of endemic regions^2,3^. In 2003, the United States had an outbreak of monkeypox, which was the first report of human monkeypox outside of Africa^4^. Currently, there is no proven, safe treatment for monkeypox virus infection. Intramuscular administration of vaccinia immune globulin (VIG) is the only approved postexposure therapy, but the efficacy remains controversial^3^.

With the advent of CRISPR–Cas (clustered regularly interspaced short palindromic repeats [CRISPR]—CRISPR-associated [Cas] systems) DNA editing technology and its many therapeutics applications, there is a unique opportunity to develop an antiviral therapy directly targeted towards monkeypox and other zoonotic poxviruses. The CRISPR–Cas system induces double-strand DNA breaks at specific genomic loci identified by single-guide RNAs (sgRNA)^5^. Although the discovery of this endonuclease is relatively new, its applications are quickly being utilized in a variety of different genome engineering and medical research applications. Recently published studies include CRISPR/Cas9-mediated control of HIV both in vitro^6–8^ and in vivo^9^. CRISPR technology has also been studied as an antiviral therapy for hepatitis viruses^10–12^, herpes viruses^13,14^, and human papillomavirus^15^.

Furthermore, the successful and safe delivery of CRISPR components to host cells has been of particular interest in regard to potential clinical therapies. Several viral vectors, including lentivirus, adenovirus, and adeno-associated virus (AAV), have been utilized as packaging and delivery vehicles for CRISPR antiviral therapeutics^16^. AAV has proven to be an effective vector for the CRISPR–Cas system as it produces a high viral titer, has broad tissue tropism, and is safe for in vivo applications since it has a low risk of integration into the host genome^17^. While CRISPR technology is often used with the Cas9 protein isolated from *S. pyogenes* (SpCas9) that is encoded by a 4.1 kb gene, this transgene is too large to be efficiently packaged with its sgRNA into the 4.7 kb packaging space of a single AAV-2 particle^18^. Alternatively, the Cas9 protein isolated from *S. aureus* (SaCas9) has a shorter coding sequence of 3.2 kb and maintains endonuclease activity that is comparable to that of SpCas9; therefore, it is a promising endonuclease to utilize with AAV vectors^18^.

In this proof of concept study, we utilized the CRISPR–SaCas9 editing system to target essential genes in orthopoxviruses, employing an AAV vector for delivery to host cells in vitro. These methods provide an avenue to translate the work to in vivo studies as AAV vectors have already been proven effective for antiviral therapies in animal models^19,20^.

## Results

### Designing CRISPR gene targets

Three essential genes were chosen to design CRISPR targets against, with three sgRNAs per gene (Table 1). The genes selected include E3L, I2L, and A17L. We used vaccinia virus (VACV) as a model organism for these experiments; however, the gene targets identified are conserved throughout several orthopoxvirus species, including monkeypox and smallpox, rendering these sequences as universal orthopoxvirus targets (Table 2). When choosing genes of interest, function and location on the viral genome were both important considerations. We ensured that these genes were located on different genomic loci to ensure optimal DNA cleavage by Cas9 and reduce possible steric hinderance. Additionally, the three genes chosen were all identified as essential genes that are required for the survival of the virus. By targeting these genes and introducing a double-stranded DNA break, we were attempting to both render these genes nonfunctional for transcription, thereby reducing the translation of viral protein, and to reduce the number of intact genomes for effective infectious viral packaging and release.

**Table 1:** Sequence of sgRNA and targeted location on VACV genome.

sgRNA name	Location on VACV genome	sgRNA sequence
E3L_1	48,179–48,152	5′-CUCCGACGAUAUUCCUCCUCGU-3′
E3L_2	48,051–48,078	5′-AAAGACUUAUGAUCCUCUCUCA-3′
E3L_3	47,827–47,854	5′-UAGCUGCAUUAUUUUUAGCAUC-3′
I2L_1	60,828–60,855	5′-AAUACAAAUAUAUCAAUAGUAG-3′
I2L_2	60,881–90,908	5′-AACCAAUACCAACCCCAACAAC-3′
I2L_3	61,026–60,999	5′-AAGUUGUACGCCGCUAUAUUUG-3′
A17L_1	125,654–125,681	5′-GUUUGUUGCAGGUAUACUGUUC-3′
A17L_2	125,761–125,788	5′-UAAGAAAUAAUAUUAAAUAUCU-3′
A17L_3	126,0420–126,069	5′-AUAAUCAUUCAUUCCUCCAUAA-3′
Neg_sgRNA	N/A	5′-AUCUAUUGUUCCGACGUAUUAU-3′

**Table 2:** Conservation of CRISPR targets among Orthopoxvirus species.

Orthopoxvirus species	Gene	Target with 100% homology		
		1	2	3
Akhmeta virus	E3L	X	X	X
	I2L	X		X
	A17L			X
Buffalopox virus	E3L	X	X	X
	I2L	X	X	X
	A17L	X	X	X
Camelpox virus	E3L	X	X	X
	I2L	X		X
	A17L	X	X	X
Cowpox virus	E3L	X	X	X
	I2L	X	X	X
	A17L	X	X	X
Ectromelia virus	E3L		X	X
	I2L	X	X	
	A17L		X	
Horsepox virus	E3L	X	X	X
	I2L	X		X
	A17L	X	X	X
Monkeypox virus	E3L		X	X
	I2L	X		
	A17L	X	X	
Orthopoxvirus Abatino	E3L	X	X	X
	I2L	X	X	
	A17L		X	X
Vaccinia virus	E3L	X	X	X
	I2L	X	X	X
	A17L	X	X	X
Variola virus	E3L	X	X	X
	I2L	X	X	X
	A17L	X	X	X

### Conservation of essential gene targets among Orthopoxvirus species

Since there are several Orthopoxviruses that can harm humans, we ensured that the gene targets chosen were conserved among several Orthopoxviruses species (Table 2). Therefore, essential genes that are present throughout Orthopoxvirus species were selected, and conserved regions within these genes were designated as CRISPR targets. These targets were confirmed in silico to have minimal off-target effects within the human genome. This gives way to the broad-spectrum use of the sgRNA targets in numerous poxvirus infections, including emerging zoonotic viruses. Furthermore, we selected genes that are involved in different aspects of the virus lifecycle and host interaction in order to design a multiplexed approach for future work in animals and ultimately in humans.

I2L is conserved in all orthopoxviruses and is a late protein that is essential for mature viral production, telomere binding, and entry into target cells. Previous research has been conducted studying the impact of the presence of this gene, demonstrating that in the absence of this protein, virions have a ~ 400-fold reduction in specific infectivity because they are unable to enter target cells^21^. This data supports the use of this gene as a promising CRISPR target to reduce the burden of VACV infection.

The gene A17L encodes a viral envelope protein that is essential for an early step in virion morphogenesis. Rodriguez et al. demonstrated that removal of this protein reduces viral yields by approximately 3 log units and morphogenesis was completely arrested at an early stage^22^. Similarly, inhibiting the translation of the full protein using CRISPR will also have a deleterious effect on the viral yield, which is supported by our data.

Because the viral gene E3L affects the host’s innate immune response, we believe that it would prove more suitable for an in vivo animal model study, and therefore, have not included it in our in vitro experimentation. Further information is discussed in the Supplemental Information.

### Determining off-target effects of CRISPR targets

As with any study using CRISPR editing technology, it is imperative to evaluate any theoretical off-target effects to ensure the CRISPR targets are safe to use. All of the potential sgRNAs that were designed were evaluated using the NCBI Basic Local Alignment Search Tool (BLAST) as well as Cas-OFFinder software^23^. According to these algorithms, there was no alignment or predicted off-target host effects for any of the potential CRISPR targets when allowing for up to two mismatches in the sgRNA sequences.

### Removing NLS from CRISPR plasmid

The CRISPR plasmid pX601-AAV-CMV::NLS-SaCas9-NLS-3xHA-bGHpA;U6::BsaI-sgRNA (a gift from Feng Zhang via Addgene, Catalog #61591) is a single vector AAV-Cas9 system containing SaCas9 and Type IIS sites to clone in an individual sgRNA. This plasmid contains N- and C-terminus nuclear localization sites (NLS); however, since poxviruses replicate in the cytoplasm, these sites were removed so the DNA would be delivered to the site of infection instead of the nucleus^24,25^. The NLS were removed using the Q5 Site-Directed Mutagenesis Kit. Successful removal of these sites and cloning of sgRNA were verified by sequencing the plasmids using Sanger sequencing^26^.

### CRISPR targets reduce VACV titer in host cells

**Figure 1:**
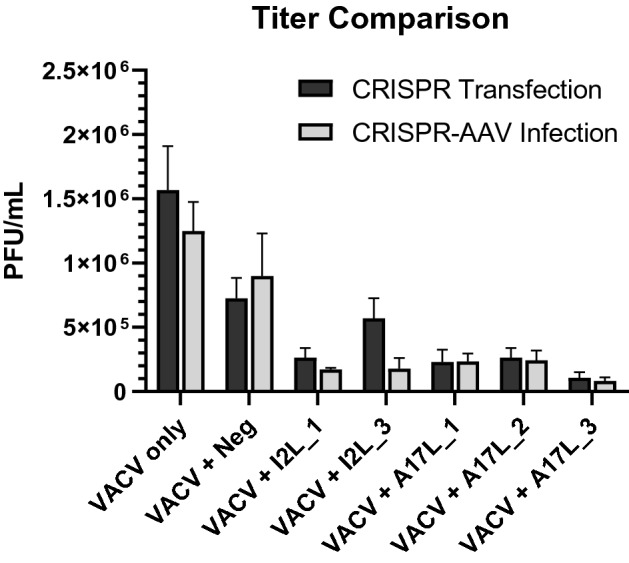
Comparison of VACV titer when transfected with CRISPR components vs. infected with CRISPR-AAV.

Before AAV production, an experiment to test the efficacies of individual CRISPR targets was conducted by directly transfecting host cells with a pAAV-SaCas9-sgRNA plasmid then infecting the cells with VACV. All samples in this experiment were tested in triplicate to determine statistical significance using a two-sided t-test comparing the VACV titer to that of the negative sgRNA and to the VACV control. We determined the effectiveness of our CRISPR targets by transfecting human embryonic kidney (HEK293) cells with plasmids encoding SaCas9 and an individual sgRNA. The cells were first transfected with the CRISPR plasmids, and after 48 h, the cells were infected with VACV at a MOI of 0.1. The cells that had been equipped with the anti-VACV targets decreased the titer of vaccinia virus, as determined by a Median Tissue Culture Infectious Dose assay (TCID_50_ assay) (Fig. 1, Table 3). The VACV titer following exposure to the negative sgRNA target was not significantly different from the VACV control titer (p = 0.09), indicating that the transfection method alone does not significantly affect VACV viability. All VACV-targeting sgRNA, except I2L_3, were significantly different from the negative sgRNA. The p value for I2L_3 was 0.52, while all other CRISPR targets had a p value that was ≤ 0.05 (Table 3).

**Table 3:** Percent vaccinia titer compared to control following transfection with CRISPR components.

Transfected Crispr target	VACV titer (PFU/mL)	% VACV compared to control	Two-sided t-test with Neg_sgRNA	Two-sided t-test with VACV control
vacv control	1.57 (± 0.342) × 10^6^	100%	*p = 0.09	–
I2L_1	2.64 (± 0.733) × 10^5^	16.82%	p = 0.05	p = 0.02
I2L_3	5.69 (± 1.58) × 10^5^	36.24%	*p = 0.52	*p = 0.06
a17L_1	2.30 (± 0.951) × 10^5^	14.65%	p = 0.05	p = 0.02
a17L_2	2.64 (± 0.733) × 10^5^	16.82%	p = 0.05	p = 0.02
a17L_3	1.07 (± 0.442) × 10^5^	6.82%	p = 0.02	p = 0.01
Neg_sgRNA	7.27 (± 1.58) × 10^5^	53.31%	–	*p = 0.09

Standard error of titer data included in parentheses. P values with an asterisk indicate the titer is not significantly different from that of Neg_sgRNA or VACV control (p ≥ 0.05).

### Successful delivery of CRISPR targets via AAV vectors

**Figure 2:**
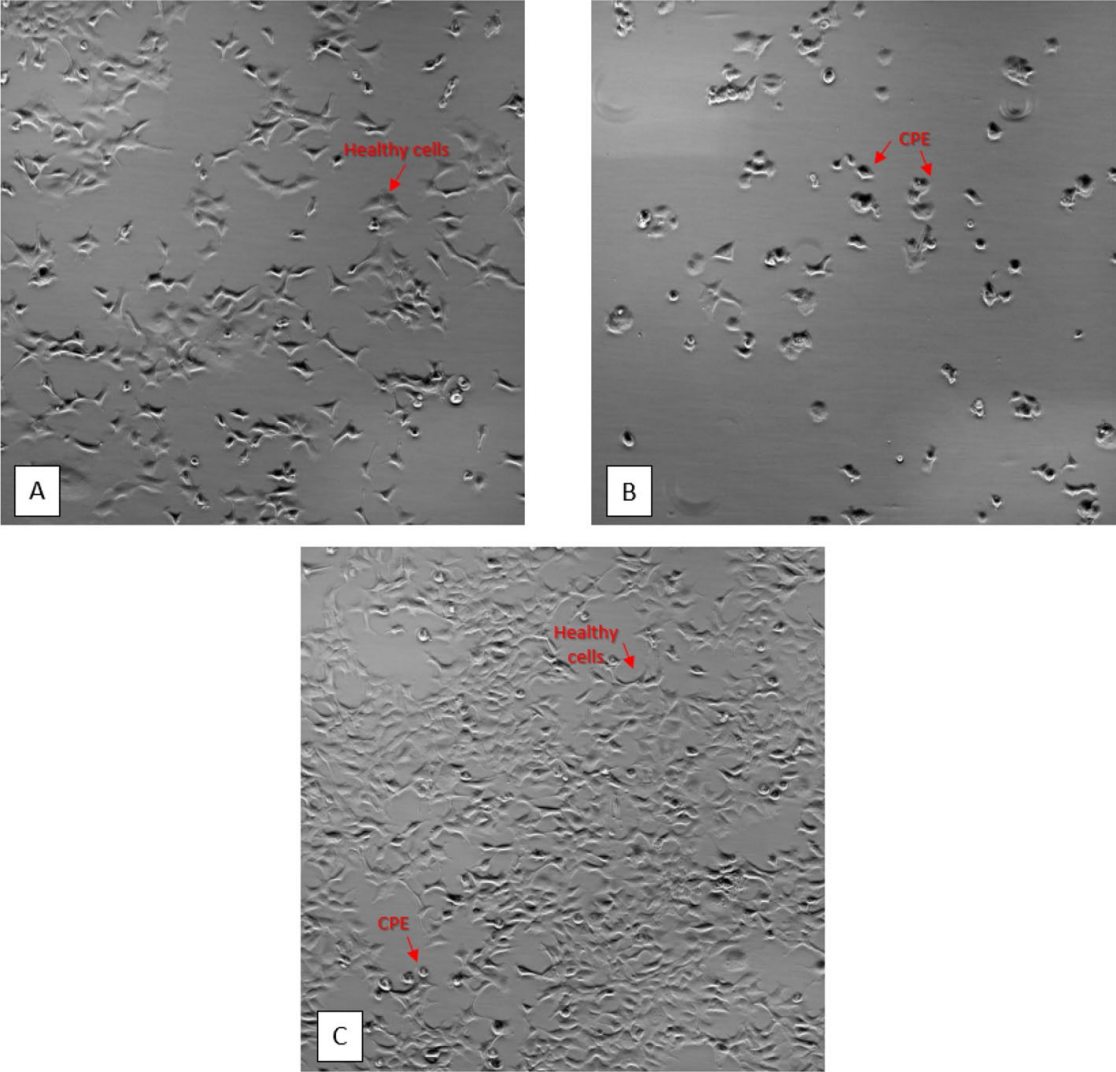
HEK293 cells following VACV exposure. Microscopy images of HEK293 host cells at 40 × magnification. (**A**) HEK293 negative control cells not infected with VACV are healthy and do not express CPE. (**B**) HEK293 positive control cells infected with VACV without CRISPR-AAV show very few healthy cells and extensive CPE. (**C**) HEK293 cells infected with both VACV and CRISPR-AAV particles have many healthy cells and few CPE.

One of the challenges with CRISPR-based therapy is providing effective, safe and targeted delivery of the CRISPR cargoes. AAV-2 proved to be a successful delivery vector for CRISPR components to target VACV infection in human cells. AAV particles were created in HEK293 cells following the protocol described below and quantified using reverse transcriptase quantitative polymerase chain reaction (RT-qPCR). AAV particles encoding for SaCas9 and single sgRNAs were delivered to HEK293 cells at a MOI of 10^4^. HEK293 cells were then subject to infection by vaccinia virus for three days. The cell lysate was collected and VACV titer was determined by performing a TCID_50_ assay. This experiment was conducted in triplicate, with three independent biological replicates to determine statistical significance of the data. Samples that were exposed to CRISPR-AAV particles had a reduction in VACV titer by up to 93% compared to the controls without CRISPR and were significantly different (two sided t-test, p ≤ 0.05) from both the negative sgRNA control and the VACV control (Fig. 1, Table 4). Comparing the values to the negative control demonstrates that the reduction in viral titer is significantly different from any effect of AAV, while comparing values to the VACV control confirms that the targets significantly reduce VACV titer. Because both these t-tests were significant for all the sgRNAs, we can confidently validate the efficacy of each of the CRISPR targets. Furthermore, HEK293 cells that were equipped with CRISPR-AAV particles demonstrated less cytopathic effects (CPE) than those infected with VACV alone (Fig. 2).

**Table 4:** Percent vaccinia titer compared to control following CRISPR-AAV exposure.

AAV Crispr target	VACV titer (PFU/mL)	% VACV compared to control	Two-sided T-test with NEG_SGRNA		Two-sided t-test with VACV control
VACV control	1.25 (± 0.306) × 10^6^	100%	*p = 0.5417	–	
I2L_1	1.71 (± 0.346) × 10^5^	13.69%	p = 0.0016	p = 0.0067	
I2L_3	2.17 (± 0.857) × 10^5^	17.41%	p = 0.0061	p = 0.0094	
a17L_1	1.98 (± 0.555) × 10^5^	15.87%	p = 0.0029	p = 0.0081	
a17L_2	2.70 (± 0.901) × 10^5^	21.62%	p = 0.0107	p = 0.0130	
a17L_3	8.78 (± 2.13) × 10^4^	7.03%	p < 0.001	p = 0.0042	
Neg_sgRNA	8.98 (± 2.24) × 10^5^	71.94%	–	*p = 0.5417	

Standard error of titer data included in parentheses. p values with an asterisk indicate the titer is not significantly different from that of Neg_sgRNA or VACV control (p ≥ 0.05).

### Determining transduction efficiency of AAV particles into host cells

Because delivery efficiency is an important factor in potential therapeutics, we identified the transduction efficiency of the AAV vector by detecting green fluorescent protein (GFP)-expressing AAV particles inside host cells. To achieve this, we inserted a green fluorescent protein (GFP)-expressing plasmid in the cargo of an AAV particle, infected HEK293 cells with the AAV-GFP vector, then determined the presence of GFP inside the cells 24 h post-infection using microscopy. The average percentage of GFP-expressing cells from the AAV-GFP vector was 75.4% with a standard error of ± 1.068, which was statistically different than the negative control without GFP (p < 0.0001). More information about this experiment is presented in Supplementary Table S1.

### Expression of SaCas9 in host cells optimized 48 h post-AAV infection

We verified that SaCas9 mRNA and protein were expressed following the delivery of CRISPR-AAV particles to HEK293 cells. To determine SaCas9 mRNA expression, we performed RT-qPCR in triplicate on samples collected 24 and 48 h post-AAV infection to confirm HEK293 cells were expressing DNA from the AAV particles. SaCas9 mRNA was expressed at both timepoints, with expression increasing at 48 h for both AAV targets tested (Table 5).

**Table 5:** SaCas9 mRNA expression.

AAV Crispr target	Expression at 24 h	Expression at 48 h
A17L_1	9.96 × 10^4^ GE/mL	2.29 × 10^5^ GE/mL
i2l_3	3.54 × 10^5^ GE/mL	6.28 × 10^5^ GE/mL

**Figure 3:**
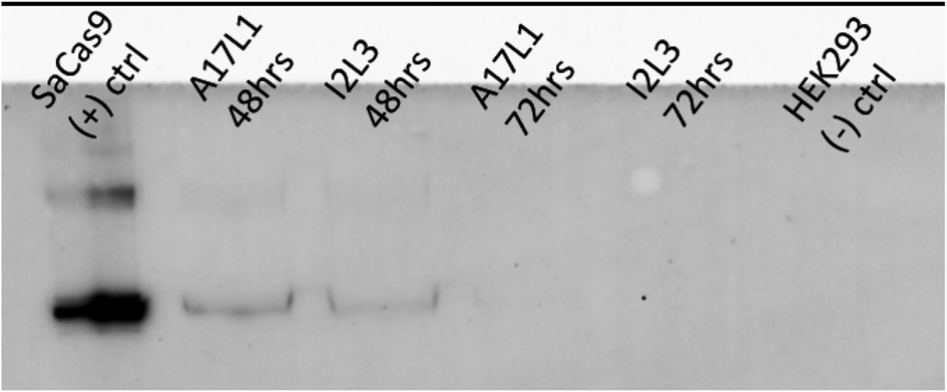
Western blot for SaCas9 protein expression. Protein expression for SaCas9 was shown in the positive control as well as in A17L and I2L targets 48 h post-infection. Protein expression was not detected for either targets at 72 h post-infection. (Image is cropped. Full-length blot is presented in Supplementary Fig. S1).

We confirmed the expression of SaCas9 protein with a Western blot assay (Fig. 3). We tested samples that were collected 48 and 72 h post-AAV infection, and the 48-h timepoint showed expression of the protein, which is consistent with the RT-qPCR data. These data demonstrate that both the mRNA and protein expression of SaCas9 from the CRISPR-AAV particles are optimized 48 h post-infection.

### Efficacy of CRISPR targets on VACV titer

The transfection of CRISPR components and the AAV delivery of the transgene for both SaCas9 and sgRNA demonstrate a significant reduction in viral titer. The resulting VACV titer following delivery of VACV-targeting sgRNA were significantly different (p ≤ 0.05) than that of the negative sgRNA, indicating that the reduced titer is not merely due to the methods of transfection or AAV infection (Tables 3, 4). Furthermore, the resulting titer for both of these CRISPR delivery methods were very similar (Fig. 1). Because the reduction in viral titer did not vary between the two methods, this demonstrates the effectiveness of AAV as a packaging and delivery vector for the CRISPR components. While both methods demonstrate the effectiveness of our CRISPR targets, the efficacy of the AAV delivery method represents the success of using AAV as a vector. Utilizing an effective vector for delivery of CRISPR components is imperative in applying these antivirals as therapeutics since there must be a safe packaging and delivery system in vivo*.*

## Discussion

In this proof of concept study, we designed CRISPR targets against VACV that also target homologous regions to other more virulent Orthopoxviruses. These targets were confirmed to successfully reduce viral titer when transfected into host cells prior to infection with VACV. Furthermore, SaCas9 and an individual sgRNA were successfully packaged into an AAV-2 vector for delivery to the host cell. We demonstrated that AAV was an effective vector to deliver CRISPR components that efficiently reduced viral titer. Not only did the CRISPR-AAV particles lower viral production, but they also had a protective effect for the host cells as they reduced the amount of CPE in the host cells following infection. Ultimately, we have developed an in vitro antiviral system using CRISPR-AAV particles to safely and successfully deliver CRISPR components, reduce viral titer, and protect host cells.

### CRISPR target efficiency

The efficiency of the CRISPR antiviral varied between the individual sgRNAs that were tested. The target against A17L_3 yielded the greatest reduction in VACV both when transfecting the CRISPR components and when delivering via the AAV vector. Although target I2L_3 was not significantly different from the negative sgRNA or the VACV control during transfection (p = 0.52, p = 0.06), the efficiency increased when using AAV to package this target (p = 0.0061, p = 0.0094). Overall, the t-test confirmed that the efficiency of all the CRISPR targets increased with the CRISPR-AAV compared to transfection alone. The p value for all the targets was ≤ 0.01 for CRISPR-AAV, while the p value for transfection was ≤ 0.05 (Tables 3, 4). Although individual sgRNA targets performed with varying efficacy, future research should be conducted to determine if there are increased effects when multiplexing several different targets.

### CRISPR-AAV particles decrease CPE

We have shown that CRISPR-AAV particles not only lower viral production, but they also have protective effects for the host cells. When delivering the CRISPR targets in an AAV vector, we observed that host cells equipped with CRISPR-AAV particles demonstrated less CPE than those infected with VACV alone (Fig. 2). This is an important distinction since it shows that some CPE are mitigated in the host cells in addition to a reduction in viral production.

### Benefits and limitations of AAV vectors for therapeutic delivery

Recombinant AAV vectors have low pathogenicity and low immunogenicity compared to other viral vectors, but their main obstacle is their limited packaging size. In this study, we were able to overcome the size limitation of AAV by utilizing SaCas9 instead of SpCas9^18,27^. An additional limitation of using AAV as an antiviral delivery vehicle is that this virus cannot replicate itself without the presence of a helper virus, thereby limiting the amount of antiviral treatment that can be provided at one time. However, this feature is in fact advantageous when considering the safety of administering it in a clinical setting. Since AAV requires a helper virus for replication, it is generally considered to be non-pathogenic^28^. An additional attribute regarding the safety of AAV is that it remains episomal once a host cell is transduced, which reduces the potential for it to integrate into the host genome^28^.

### Overcoming limitations in CRISPR antivirals

While the CRISPR–Cas9 system has successfully been used for a variety of antiviral treatments, there remains limitations to be considered before employing it in clinical trials. One of the most prevalent concerns with CRISPR technology is the potential for off-target effects which may induce gene mutations or chromosomal translocations^29^. However, there have been several methods to attempt to reduce off-target effects such as dimerization dependent RNA-guided FokI-dCas9 nucleases (RFNs), truncated guide RNAs (tru-gRNAs), and paired Cas9 nickase^30^. We have thoroughly analyzed the potential for off-target effects in our CRISPR targets using two different predictive algorithms to ensure the targets are not only effective but are also safe to use.

Another limitation to the CRISPR technology is the development of escape virus variants. Because of non-homologous end joining (NHEJ) repair, the poxvirus may mutate in one of the locations of the sgRNA targets, causing this CRISPR target to become ineffective. It has also been shown that CRISPR–Cas9 could generate mutant viruses able to resist to Cas9/sgRNA by causing DNA repair in host cells^31,32^. Solutions have been developed to contend with this escape mechanism which include modifying sgRNA, reprogramming Cas9 nuclease, and suppressing NHEJ activity^33^. We have mitigated the potential for escape mutants by using redundancy when developing our CRISPR targets. Since we have multiple sgRNAs targeting one gene and since we have targets for more than one gene, if the virus becomes resistant to one of the targets, there is a library of other effective targets that can be used.

### Further research

In the present work, we only tested individual CRISPR targets; however, multiplexing the targets within a single AAV vector provides the potential for a greater reduction in viral infectivity. This cocktail approach has already proven effective in previous work, and it was shown that AAV vectors could accommodate up to four sgRNAs^9^. By utilizing a multiplex approach for CRISPR targets, the potential for escape virus variants that can evade the CRISPR endonuclease is alleviated. While we have shown that individual CRISPR targets can successfully reduce the viral titer of VACV, there may be a greater effect when the virus is exposed to multiple dsDNA breaks.

Since we have shown the efficacy of this antiviral therapy in vitro, this work gives way to investigate the results of these procedures in vivo*.* AAV vectors have already been utilized in numerous animal studies and have proven to be a safe and effective delivery mechanism. Therefore, the results of our work, coupled with previous research using CRISPR-AAV particles as an antiviral therapy, provide indication for the success of this method in an in vivo study.

Further investigation should also be done to test the optimal timing of antiviral delivery following VACV infection. A series of experiments to determine the efficacy of each target over several timepoints would provide insight into how the effectiveness of this antiviral may change over time and through the course of an infection. Currently, our experiments have identified the effect of the CRISPR-AAV particles when administered prior to VACV exposure. It would be valuable to evaluate the efficacy of CRISPR-AAV transduction following VACV infection as a post-infection dose experiment to determine if this method is effective as a prophylactic or post-exposure treatment. These experiments are important to refine this proof of concept antiviral treatment when transitioning it into a potential clinical therapeutic.

## Materials and methods

### Design of sgRNAs

We used Geneious 9.0.5 software (https://www.geneious.com) to construct sgRNAs against the three essential gene targets. To design the sgRNAs, we specified 5′-NNGRRT-3′ as the PAM sequence for optimal on-target cleavage by SaCas9. When determining which sgRNA to choose for a particular gene target, the targets with the highest on-target activity were prioritized. Off-target effects of these sgRNAs were determined by running the Basic Local Alignment Search Tool (BLAST) as well as Cas-OFFinder software^23^. The Cas-OFFinder algorithm was set to evaluate sequences with up to two mismatches in the sequence within the human genome. It was previously determined that when crRNA sequences contained two disruptive mismatches, less than 5% of the crRNA were able to effectively cleave their target^34^. Therefore, two mismatches were used to determine potential off-target effects. BLAST identified the closest organisms that aligned with the CRISPR targets.

### AAV-SaCas9 plasmid preparation

A pair of oligonucleotides with a 5′—CACC overhang and a 3′—AAAC overhang were synthesized by IDT for each of the target sites (Table 6). Each complementary oligo pair was phosphorylated and annealed together by combining 100 μM of each oligo pair with T4 polynucleotide kinase (PNK) and T4 Ligation Buffer then running them in a thermocycler at 37 °C for 30 min., 95 °C for 5 min, and ramping down to 25 °C at 5 °C/min. The oligos were then cloned into pX601-AAV-CMV::NLS-SaCas9-NLS-3xHA-bGHpA;U6::BsaI-sgRNA (A gift from Feng Zhang via Addgene, Catalog #61591)^18^. The plasmid was digested with BsaI restriction enzyme sites and the oligo pairs were ligated into the plasmid with T7 ligase and ran on a thermocycler for 6 cycles of 37 °C for 5 min and 21 °C for 5 min. The plasmid was then treated with PlasmidSafe exonuclease to digest any residual linearized DNA. The cloned plasmids were transformed into Stbl3 cells, and the DNA was purified using GeneJET Plasmid Miniprep Kit (Thermo Scientific, Catalog #K0503). Cloning was verified via Sanger Sequencing performed by Eurofins Genomics.

**Table 6:** sgRNA-targeting oligonucleotide sequences.

Oligo name	Direction	DNA sequence
I2L1_sgRNA_Top	Sense	5′-caccgAATACAAATATATCAATAGTAG-3′
I2L1_sgRNA_Bottom	Antisense	5′-aaacCTACTATTGATATATTTGTATTc-3′
I2L2_sgRNA_Top	Sense	5′-caccgAACCAATACCAACCCCAACAAC-3′
I2L2_sgRNA_Bottom	Antisense	5′-aaacGTTGTTGGGGTTGGTATTGGTTc-3′
I2L3_sgRNA_Top	Sense	5′-caccgAAGTTGTACGCCGCTATATTTG-3′
I2L3_sgRNA_Bottom	Antisense	5′-aaacGCAAATATAGCGGCGTACAACTTc-3′
A17L1_sgRNA_Top	Sense	5′-caccgGTTTGTTGCAGGTATACTGTTC-3′
A17L1_sgRNA_Bottom	Antisense	5′-aaacGAACAGTATACCTGCAACAAACc-3′
A17L2_sgRNA_Top	Sense	5′-caccgTAAGAAATAATATTAAATATCT-3′
A17L2_sgRNA_Bottom	Antisense	5′-aaacAGATATTTAATATTATTTCTTAc-3′
A17L3_sgRNA_Top	Sense	5′-caccgATAATCATTCATTCCTCCATAA-3′
A17L3_sgRNA_Bottom	Antisense	5′-aaacTTATGGAGGAATGAATGATTATc-3′
Neg_sgRNA_Top	Sense	5′-caccgATCTATTGTTCCGACGTATTAT-3′
Neg_sgRNA_Bottom	Antisense	5′-aaacATAATACGTCGGAACAATAGATc-3′

Because poxviruses replicate in the cytoplasm, it was necessary to remove the N- and C-terminus nuclear localization sites (NLS) on the plasmid so the DNA would be delivered to the site of infection instead of the nucleus^24,25^. The NLS were removed using the Q5 Site-Directed Mutagenesis Kit (New England Biolabs, Catalog #E0554S) following the manufacturers protocol. Mutagenesis primers were developed using NEBaseChanger Software to optimize the use of the Q5 SDM Kit (Table 7). Cloning was verified with Sanger Sequencing via Eurofins Genomics^26^. After confirmation of sgRNA target insertion and deletion of NLS from the pAAV-SaCas9-sgRNA plasmid, large stocks of the plasmid DNA were produced using QIAGEN Plasmid Maxi Kit according to the manufacturer’s protocol (QIAGEN, Catalog #12165).

**Table 7:** Q5 SDM primers.

Primer name	Melting temperature (°C)	Amplification temperature (°C)	DNA sequence
N-Terminus Fwd	70	71	5′-AAGCGGAACTACATCCTGGGC-3′
N-Terminus Rev	74	71	5′-GGCCATGGTGGGACCGGT-3′
C-Terminus Fwd	62	58	5′-GGATCCTACCCATACCATG-3′
C-Terminus Rev	57	58	5′-GCCCTTTTTGATGATCTG-3′

### AAV production and quantification

AAV particles were produced from the pAAV-SaCas9-sgRNA plasmid via a triple transfection method using the AAV-2 Helper-Free Packaging System (Cell Biolabs, Catalog #VPK-402). A calcium phosphate transfection kit (Invitrogen, Catalog #K2780-01) was used to transfect Cell Biolabs 293AAV cells with 10 μg pAAV-RC2, 10 μg pHelper, and 10 μg pAAV-SaCas9-sgRNA. Following transfection, cells were washed with PBS and fresh DMEM-10 was added. After incubating the transfected cells at 37 °C, 5% CO_2_ for 48 h, the cells and media were collected from the flask with a cell scraper and placed in a 15 mL conical tube. The cells then underwent 4 freeze/thaw cycles using an ethanol-dry ice bath and a 37 °C water bath. The tube was centrifuged at 10,000 × *g* for 10 min at room temperature, and the supernatant containing the AAV particles was collected and stored at − 80 °C.

AAV particles were quantified using qPCR targeting the SaCas9 gene to determine the genomic equivalents per mL (GE/mL). AAV DNA was isolated using the Zymo Viral DNA/RNA Miniprep Kit (Zymo, #D6005) following the standard protocol. The primers and probe to detect SaCas9 were designed using Geneious 9.0.5 software. The forward primer sequence is 5′-AGGGCAGAATCAGCAAGACC-3′ and the reverse primer sequence is 5′-CACCAGGTTCCGGTTGATGA-3′. A TaqMan probe was designed with the sequence (5′-(6-FAM) CTGCTGGAAGAACGGGACAT (MBGQ)-3′. We used 500 nM of each primer and 250 nM of the TaqMan probe with Platinum Quantitative PCR SuperMix-UDG (Invitrogen, #11730-025) and 5 μL of DNA for the qPCR reaction mixture. The reaction was run on a thermocycler at 50 °C for 2 min followed by 95 °C for 2 min, then 35 cycles of 95 °C for 15 s. and 60 °C for 1 min. The quantity of AAV particles was determined by converting C_T_ values into GE/mL according to the qPCR standard curve.

### VACV production

Vaccinia virus (ATCC, Catalog #VR-1536) was propagated in Vero cells (ATTC, Catalog #CCL-81) when the host cells grew to 80% confluence. The virus was thawed in a 37 °C water bath and diluted 1:10 in EMEM. The media was removed from the Vero cells and 2.5 mL virus dilution was added to each T-75 cm^2^ flask. The flasks were incubated at 37 °C, 5% CO_2_ for 2 h, then 10 mL EMEM + 2% FBS was added to the flasks. The cells were monitored for the development of cytopathic effects (CPE), and the virus was harvested 2 days post-infection when the infection reached 75–100% of cells. The virus was harvested by scraping the cells into the medium and quick-freezing in liquid nitrogen vapor. The viral titer was determined by performing a TCID_50_ assay, and these values were converted to PFU/mL using the Spearman–Kärber statistical method. The calculations for this conversion have previously been described by Lei et al.^35^.

### Transfection of pAAV-SaCas9-sgRNA and infection with VACV in HEK293 cells

A calcium phosphate transfection kit (Invitrogen, Catalog #K2780-01) was used to transfect HEK293 cells with 10 μg pAAV-SaCas9-sgRNA. Following transfection, cells were washed with PBS and fresh DMEM-10 was added, then the cells were incubated at 37 °C, 5% CO_2_ for 48 h. After incubation, the media was aspirated, and 2.5 mL of VACV was added at a MOI of 0.1. The cells were incubated at 37 °C, 5% CO_2_ for 2 h, then 10 mL of DMEM-2 was added and the incubation continued for 3 days. The cells lysate was harvested by scraping the cells into the media using a cell scraper and immediately freezing in liquid nitrogen. The viral titer was determined by performing a TCID_50_ assay, and the titer of cells with CRISPR components was compared to the positive control with only VACV.

### VACV/AAV coinfection in HEK293 cells

HEK293 cells were infected with CRISPR-encoding AAV particles to allow cells to express SaCas9. AAV particles were added to the cells at a MOI of 10^4^ GE/cell and incubated at 37 °C, 5% CO_2_ for 24 h. The cells were then infected with 1 mL VACV at a MOI of 0.1 GE/cell and incubated at 37 °C for 1.5 h, then 4 mL DMEM-2 was added and the incubation continued for 3 days before harvesting the lysate. The cells lysate was harvested by scraping the cells into the media using a cell scraper and immediately freezing in liquid nitrogen. The viral titer was determined by performing a TCID_50_ assay, and the titer of lysates with CRISPR components was compared to the positive control with only VACV to determine the efficacy of the CRISPR/Cas9 system on the VACV titer.

### Transduction efficiency with AAV-GFP

AAV-GFP particles were produced from a pAAV-GFP plasmid (Addgene #32395) using the triple transfection method described previously. HEK293 cells were infected with AAV-GFP particles at a MOI of 1 × 10^5^ viral genomes/cell and incubated at 37 °C, 5% CO_2_ for 24 h. Transduced HEK293 cells were imaged using an Olympus IX83 microscope under GFP fluorescence to determine the percentage of cells with effective transduction by AAV-GFP particles^36^.

### SaCas9 RT-qPCR

mRNA was extracted from cell lysates 24 and 48 h post-AAV infection using the Dynabeads mRNA Direct Kit (Thermo Fisher, Catalog #61011) following the manufacturer’s protocol. The primers and probe to detect SaCas9 were designed using Geneious 9.0.5 software. The forward primer sequence is 5′-AGGGCAGAATCAGCAAGACC-3′ and the reverse primer sequence is 5′-CACCAGGTTCCGGTTGATGA-3′. A TaqMan probe was used with the sequence (5′-(6-FAM) CTGCTGGAAGAACGGGACAT (MBGQ)-3′. The TaqMan RNA-to-C_T_ 1-Step Kit (Invitrogen, Catalog #4392653) was used to reverse transcribe the RNA into cDNA according to the standard protocol. We used 500 nM of each primer and 250 nM of the TaqMan probe in the qPCR reaction mix. The reaction was run on a thermocycler at 48 °C for 15 min. followed by 95 °C for 10 min., then 40 cycles of 95 °C for 15 s. and 60 °C for 1 min. The expression of SaCas9 mRNA was determined by converting C_T_ values into genomic equivalents per mL (GE/mL) according to the standard curve.

### Western Blot

HEK293 cells were plated with 4.2 × 10^6^ cells in each T-75 cm^2^ flask with 10 mL DMEM-10. Immediately after seeding the cells, they were infected with AAV particles at a MOI of 1 × 10^4^. The cells were incubated at 37 °C with 5% CO_2_. The cell lysate was collected 48 h and 72 h post-infection by washing the cells with ice-cold PBS, then adding 500 μL lysis buffer (20 mM HEPES pH 7.5,100 mM KCl, 5 mM MgCl2, 1 mM DTT, 5% glycerol, 0.1% Triton X-100, supplemented with Halt Protease Inhibitor Cocktail [Thermo Fisher, Catalog #78429]). The cells were scraped off the flask, and the cell suspension was transferred into a pre-cooled microcentrifuge tube. The cells were then incubated at 4 °C with constant agitation, followed by centrifugation at 12,000 RPM for 20 min at 4 °C. The tubes were placed on ice, and the supernatant was transferred into a fresh microcentrifuge tube. The cell lysates were combined with NuPAGE LDS sample buffer (Invitrogen, Catalog #NP0008) and denatured at 95 °C for 5 min. prior to running them on an SDS-PAGE 4–12% Bis–Tris gel with protein standards (Bio-Rad, Catalog #1610374). The SDS-PAGE gel was transferred onto a Western Blot using a BioRad Trans-Blot Turbo transfer system at 1.3 A and 25 V for 10 min. We used 7% milk in TBST for the blocking buffer. The primary antibody used was Mouse SaCas9 monoclonal antibody [6H4] (EpiGentek, Catalog #A-9001), and the secondary antibody was Goat anti-Mouse IgG H&L [FITC] antibody (Abcam, Catalog #AB6785). The positive control used was purified SaCas9 nuclease protein (Applied Biological Materials, Catalog #K044), and the negative control was HEK293 cells.

### Statistical analysis

All statistical analysis was performed using SAS Studio 3.8 software^37^. Statistical significance was defined as a p value ≤ 0.05.

## Supplementary information

Supplementary Information.

## Data Availability

The datasets generated during and analyzed during the current study are available from the corresponding author on reasonable request.
