# Supplementary material for: CRISPR/Cas9 as an antiviral against Orthopoxviruses using an AAV vector
Source: Sci Rep. 2020 Nov 9;10:19307. doi: 10.1038/s41598-020-76449-9 (PMC7653928; doi:10.1038/s41598-020-76449-9)

**Supplementary Information**

**CRISPR/Cas9 as an antiviral against Orthopoxviruses using an AAV vector**

**Cathryn M. Siegrist, Sean M. Kinahan, Taylor Settecerri, Adrienne C. Greene, Joshua L. Santarpia**

## **Supplementary Discussion: sgRNA targets removed from present *in vitro* study**

The viral gene E3L inhibits the innate immune system as it antagonizes the interferon system. This gene has been shown to be essential to viral pathogenicity in VACV (27). E3L interferes with several cellular pathways, promotes cellular growth, and impairs antiviral activity and resistance to apoptosis. Although this gene is essential to the virus lifecycle and proves to be a promising CRISPR target, we did not include it in this *in vitro* study. Since this viral gene affects the host’s innate immune response, we believe that it would prove more effective in an *in vivo* animal model study. Despite excluding this target in our experimental work, the sgRNA sequence data were included in this manuscript as they are relevant to future research for *in vivo* studies.

The I2L_2 sgRNA target was also removed from further experimentation after obtaining low yields following AAV production of this target, and therefore is not included in the evaluation of this study.

**Supplementary Table S1: AAV transduction efficiency.** % GFP expression determined by dividing GFP-expressing cells by the total number of cells in present microscope field of view.

| Sample | % GFP expression | Two-sided t-test with (+) control | Two-sided t-test with (-) control | |
| --- | --- | --- | --- | --- |
| A549-GFP (+) control | 63.2 (± 1.078) % | - | | P < 0.0001 |
| AAV-GFP infected HEK293 cells | 75.4 (± 1.068) % | P = 0.0008 | | P < 0.0001 |
| HEK293 (-) control | 0% | P < 0.0001 | | - |

**Supplementary Figure S1: Western blot for SaCas9 protein expression.** Full length Western blot. Cropped blot presented in Fig 3 of the main text.


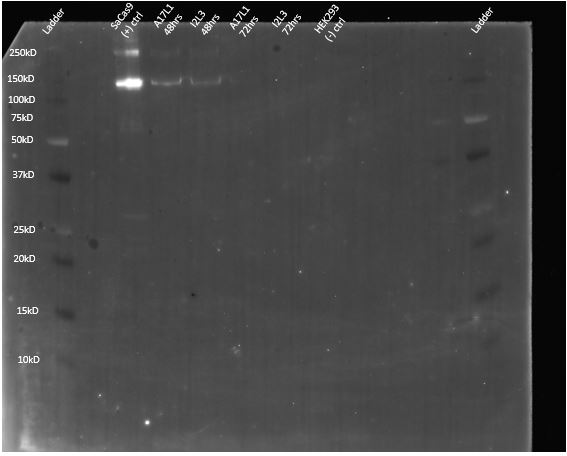

Supplement: Supplementary file 1 — Supplementary Information. [file 41598_2020_76449_MOESM1_ESM.docx]
